# *Brucella abortus* Rough-Type Mutant Induces Ferroptosis and More Oxidative Stress in Infected Macrophages

**DOI:** 10.3390/pathogens12101189

**Published:** 2023-09-23

**Authors:** Hai Hu, Guangdong Zhang, Mingxing Tian, Xiang Guan, Yi Yin, Chan Ding, Shengqing Yu

**Affiliations:** 1 Shanghai Veterinary Research Institute, Chinese Academy of Agricultural Sciences (CAAS), Shanghai 200241, China; wuhusihaixh@163.com (H.H.); gdzhang@shvri.ac.cn (G.Z.); tianmx530@126.com (M.T.); guanxiang1978@163.com (X.G.); yinyisonia@126.com (Y.Y.); 2 Veterinary Bio-Pharmaceutical, Jiangsu Key Laboratory for High-Tech Research and Development of Veterinary Biopharmaceuticals, Jiangsu Agri-Animal Husbandry Vocational College, Taizhou 225300, China

**Keywords:** *Brucella*, macrophage, oxidative stress, ferroptosis

## Abstract

*Brucella* is an intracellular parasitic bacterium that uses multiple strategies to evade the host’s defense mechanisms. However, how *Brucella* manipulates the host-induced oxidative stress and relevant biological processes are still poorly understood. In this study, a comparative transcriptome assay of macrophages infected with *Brucella abortus* S2308 and its rough mutant RB14 was performed to investigate the differentially expressed genes which might be associated with the pathogenic mechanism of *Brucella*. Our results showed that numerous host pro-oxidative and antioxidative stress genes were differentially expressed in macrophages infected with *B. abortus* S2308 and mutant RB14 at 4, 8, 24, and 48 h post-infection. Interestingly, we found that several ferroptosis-associated genes were differentially expressed during *B. abortus* RB14 infection. Moreover, we found that the rough mutant RB14-induced macrophage death was associated with reduced levels of host glutathione and glutathione peroxidase 4, together with increased free iron, lipid peroxidation, and ROS, all of which are important hallmarks of ferroptosis. The ferroptosis occurring during infection with RB14 was reduced by treatment with the inhibitor ferrostatin-1. However, *B. abortus* S2308 infection did not induce these hallmarks of ferroptosis. Taken together, our results demonstrate that ferroptosis is involved in rough *B. abortus* infection. Investigating how *Brucella* manipulates oxidative stress and ferroptosis in its host will be helpful to clarify the pathogenicity of *B. abortus*.

## 1. Introduction

Brucellosis is a serious zoonotic infectious disease caused by *Brucella*, a facultatively intracellular bacterial pathogen. *Brucella* has no classical virulence factors, such as invasive proteases, exotoxins, capsules, virulence plasmids, etc. The virulence of *Brucella* relies on its ability to evade the host’s immune system and survive within its macrophages. *Brucella* also uses its own components to regulate host-cell-related biological processes to promote the infection process. For example, *Brucella* uses the type IV secretion system (T4SS) to secrete Btp1 protein, which competes with downstream molecules of the toll-like receptors, allowing it to evade the innate immune response [1]. Another effector protein, BtpB, secreted by *Brucella* T4SS, also interferes with the immune function of the MyD88 pathway [2]. *Brucella* also uses the type IV secretion system (T4SS) to secrete TcpB protein, which affects endoplasmic reticulum stress, allowing it to evade the innate immune response and facilitating its intracellular survival [3,4]. Research has shown that *Brucella* induces host cell apoptosis and autophagy, which facilitates its spread and diffusion [5,6]. However, there are few studies on how *B. abortus* regulates the oxidative stress genes and relevant biological processes in host cells. Therefore, screening and evaluating the oxidative stress genes and related biological processes during *B. abortus* infection should be helpful to clarify the novel pathogenic mechanism of *B. abortus*.

*Brucella abortus* strain 2308 is a pathogenic strain affecting cattle and humans. RB14, a rough mutant strain lacking the O-antigen of lipopolysaccharide (LPS), is an artificially constructed derivative from the parental *B. abortus* strain S2308. *B. abortus* S2308 has evolved many remarkable strategies to evade the host immune response, which allows the establishment of chronic infections, whereas rough mutant RB14 cannot survive in macrophages. Rough *Brucella* RB14 infection also causes the death of macrophages [7]. In this study, we compared the transcriptomes of the mouse macrophage cell line RAW 264.7 infected with the *B. abortus* S2308, or the rough mutant RB14 at 4, 8, 24, and 48 h post-infection (hpi) to clarify the underlying pathogenic mechanism of *Brucella*.

Our comparative transcriptomic analysis showed that a large number of genes were differentially expressed in RAW 264.7 macrophages infected with *B. abortus* S2308 and its rough mutant RB14. We focused on those genes related to oxidative stress and programmed cell death, including ferroptosis. We then confirmed the transcriptome data with real-time PCR, which was consistent with the transcriptome data for most of the genes tested. Importantly, our study showed that *Brucella* rough mutant RB14 but not *B. abortus* S2308 induced macrophage ferroptosis in the early stage of the infection. These newly identified differentially expressed genes (DEGs) and the novel form of programmed cell death associated with *Brucella* infection should allow us to clarify the survival and replication strategies of *Brucella* within macrophages.

## 2. Materials and Methods

### 2.1. Reagents

P53 and α-tubulin antibodies were purchased from Cell Signaling Technology (Danvers, MA, USA). ACSL4, SLC7A11, and NOX2 antibodies were purchased from Abclonal (Wuhan, China). Horseradish peroxidase (HRP)-conjugated IgG (H + L) secondary antibodies and CellROX Green Reagent were purchased from Thermo Scientific (Waltham, MA, USA). The Gpx4 antibody was obtained from Abcam (Cambridge, MA, USA). The Cytotox 96 nonradioactive cytotoxicity assay kit was purchased from Promega (Fitchburg, USA). Erastin was purchased from MCE (New York, NY, USA). The Lipid Peroxidation (MDA) Assay Kit, and GSSG/GSH quantification kits were purchased from Beyotime (Shanghai, China). Hoechst 33342 and one-step RT-qPCR kits were purchased from Sangon Biotech (Shanghai, China). Ferrostatin-1 (Fer-1) was purchased from Solarbio Science & Technology (Beijing, China). Precast Gel was purchased from Tsingke Biotechnology (Beijing, China). ChamQ Universal SYBR qPCR master mix was purchased from Vazyme Biotech (Nanjing, China). FerroOrange was purchased from Dojindo Molecular Technology (Kyushu, Japan). Gentamicin and other chemicals were from Sangon Biotech (Shanghai, China). All drug concentrations are expressed as the final molar concentration in working buffer.

### 2.2. Bacterial Strains and Cell Lines

The virulent *B. abortus* S2308 strain was purchased from American Type Culture Collection (ATCC), and rough mutant RB14 was constructed in our laboratory [8]. *B. abortus* were cultured in tryptic soy broth (Difco, BD, Franklin Lakes, NJ, USA) or tryptic soy agar (TSA) at 37 °C with 5% CO_2_. RAW264.7 murine macrophages were purchased from ATCC and cultured in Dulbecco’s Modified Eagle Medium (DMEM; Hyclone, Logan, UT, USA) with 10% fetal bovine serum (FBS; Gibco, ThermoScientific) at 37 °C with 5% CO_2_.

### 2.3. Sample Collection and Preparation

RAW264.7 cells were seeded on 6-well plates at 2 × 10^6^ cells per well, 24 h prior to infection. In order to ensure the similar invasion of *Brucella*, cells were infected with *B. abortus* S2308 and rough mutant RB14 in triplicate wells of the 6-well plates at a multiplicity of infection (MOI) of 1000 and 60 by centrifuging bacteria onto cells at 400 g for 5 min at room temperature. Following 1 h of incubation at 37 °C in an atmosphere containing 5% CO_2_, the cells were washed twice with PBS to remove extracellular bacteria and incubated for an additional 1 h in medium supplemented with 100 μg/mL gentamicin to kill extracellular bacteria. Samples were collected at 4, 8, 24, and 48 hpi for further RNA extraction.

### 2.4. RNA Isolation, Library Preparation, and Sequencing

RNA degradation and contamination were monitored on 1% agarose gels. RNA purity was checked using the NanoPhotometer spectrophotometer (IMPLEN, Westlake Village, CA, USA). RNA concentration was measured using the Qubit RNA Assay Kit in Qubit 2.0 Fluorometer (Life Technologies, Carlsbad, CA, USA). RNA integrity was assessed using the RNA Nano 6000 Assay Kit of the Bioanalyzer 2100 system (Agilent Technologies, Santa Clara, CA, USA). The clustering of the index-coded samples was performed on a cBot cluster generation system using the TruSeq PE Cluster Kit v3-cBot-HS (Illumina, San Diego, CA, USA) according to the manufacturer’s instructions. After cluster generation, the libraries were sequenced on an Illumina Hiseq 2500 platform and 125 bp paired-end reads were generated.

### 2.5. Data Analysis

Raw data (raw reads) of fastq format were firstly processed through in-house perl scripts. In this step, clean data (clean reads) were obtained by removing reads containing adapter, reads on containing ploy-N, and low-quality reads from raw data. At the same time, the Q20, Q30, and GC content of the clean data were calculated, and normalized to untreated mock controls. All the downstream analyses were based on the clean data with high quality. Cuffdiff provides statistical routines for determining differential expression in digital transcript or gene expression data using a model based on the negative binomial distribution [9]. Transcripts with an adjusted *p*-value < 0.05 were assigned as differentially expressed.

### 2.6. GO Enrichment and Differential Expression Analysis

Gene ontology (GO) analysis for the biological processes was performed to identify the biological function classification of the genes, which describes properties of genes and their products. GO enrichment analysis of differentially expressed genes was implemented using the GOseq R package, in which gene length bias was corrected. GO terms with a corrected *p*-value less than 0.05 were considered significantly enriched by differential expressed genes.

### 2.7. Cell Infection and Survival Assays

Cells were seeded on 24-well plates at 3 × 10^5^ cells per well or 6-well plates at 2 × 10^6^ cells, 24 h prior to infection. Cells were infected with *B. abortus* S2308 and rough mutant RB14. Following 1 h of incubation at 37 °C in an atmosphere containing 5% CO_2_, the cells were washed twice with PBS to remove extracellular bacteria and incubated for an additional 1 h in medium supplemented with 100 μg/mL gentamicin to kill extracellular bacteria. To monitor *B. abortus* intracellular survival, the infected cells were lysed with 0.25% Triton X-100 in PBS at specific time points, and serial dilutions of lysates were rapidly plated onto TSA to enumerate colony forming units (CFUs).

### 2.8. Lactate Dehydrogenase Release Assay

Macrophages were seeded in 96-well plates and infected with S2308 and RB14 as described above. The supernatants were analyzed for lactate dehydrogenase (LDH) level using the CytoTox 96 LDH-release assay as per the manufacturer’s instructions. Percentage LDH release was calculated as 100 × ((Experimental LDH Release—Culture Medium Background)/(Maximum LDH Release—Culture Medium Background)).

### 2.9. RNA Extraction and Quantitative Real-Time PCR

Total RNA was isolated from RAW 264.7 cells (uninfected or infected with respective *B. abortus* strain) at different time points using the TRIzol reagent (Ambion, Carlsbad, CA, USA); samples were performed strictly to the manufacturer’s instructions. Genomic contamination was removed with the Turbo DNA-free kit (Ambion, Foster, CA, USA). The resulting RNA was reverse transcribed with the PrimeScript RT reagent kit (TaKaRa, Dalian, China) to produce a cDNA template. The Universal SYBR master mix was used for real-time PCR, according to the manufacturer’s instructions. A total of 1 µL of cDNA, 0.5 µL of forward or reverse primer (10 µM), 8 µL of nuclease-free water and 10 µL of 2× qPCR master mix were added and mixed. The reaction was performed on a Mastercycler ep Realplex system (Eppendorf, Hamburg, Germany). The cycling parameters were as follows: 95 °C for 2 min, 40 cycles of 95 °C for 15 s, and 60 °C for 1 min. Each gene was tested in triplicate and the β-actin gene was used as the internal control. The relative transcription level of each gene was calculated using the 2^-*∆∆*Ct^ method. The primers are listed in Table S2.

### 2.10. Immunoblot Analysis

At the indicated times, cells were lysed in western blot lysis buffer on ice for 15 min and spun at 16,000× *g* for 15 min to pellet the insoluble fraction. Soluble fractions were used for immunoblot assays. Cytosolic extracts were separated using polyacrylamide gel, transferred to nitrocellulose membranes (Millipore, Boston, MA, USA). The membranes were then blocked for 1 h in Tris-buffered saline (TBS) solution containing 0.1% Tween-20 (TBST) and 5% non-fat milk, and incubated overnight at 4 °C with primary antibodies (1:1000 dilution). Following overnight incubation, the membranes were washed with TBST and incubated with HRP-conjugated IgG (H + L) secondary antibodies (1:10,000 dilution) at room temperature for 1 h and washed three times with TBST. The protein bands were developed using ECL reagent, visualized using a Tanon 5200 automatic chemiluminescence image analysis system (Tanon, Shanghai, China), and quantified using the ImageJ software 1.0 (National Institutes of Health, Rockville, MD, USA).

### 2.11. Immunofluorescence Assay

RAW264.7 cells were cultured on 15 mm diameter glass coverslips (Thermo Scientific) in a 24-well plate and infected with *B. abortus* RB14, as described above. At the designated time points, the cells were washed twice with PBS and incubated with FerroOrange in DMEM at a final concentration of 1 μM. The cells were incubated for 20 min at 37 °C, then the coverslips were incubated with Hoechst 33342 staining solution for 10 min at 37 °C to stain DNA. Finally, the coverslips were mounted on glass slides with Eukitt quick-hardening mounting medium (Sigma-Aldrich, Burlington, MA, USA) and observed under laser scanning confocal microscopy (Nikon D-Eclipse C1, Tokyo, Japan) [10]. The assay was performed in triplicate.

### 2.12. Lipid Peroxidation Assay

The lipid peroxidation was determined by detection of the malondialdehyde (MDA) concentration in the cell lysates, which was performed strictly to the manufacturer’s instructions (Beyotime, Shanghai, China). For MDA detection, the thiobarbituric acid included in the kit was added to the supernatants of the cell homogenate to form a TBA-MDA mixture, which was then examined spectrophotometrically at 535 nm. All assays were performed with three independent replicates.

### 2.13. Measurement of ROS

RAW264.7 cells were plated in a 6-well plate. At designated time points, RAW264.7 cells were resuspended and incubated with CellRox Green in DMEM at a final concentration of 5 μM. The cells were incubated for 30 min at 37 °C, inactivated in 4% (*w*/*v*) formaldehyde for 15 min at 37 °C, and resuspended in PBS. ROS were detected using a NovoCyte flow cytometer.

### 2.14. GSH Assay

The intracellular levels of reductive GSH were determined using a GSSG/GSH quantification kit (Beyotime, Shanghai, China) to detect reductive GSH concentrations in the cell lysates, strictly according to the manufacturer’s instructions. The experiment was performed with three independent replicates.

### 2.15. Statistical Analysis

All data were imported into GraphPad Prism 8 (Graph Pad Software, Boston, MA, USA) for analysis. The differences in the data were compared using unpaired two-tailed t-tests or two-way ANOVAs with the Sidak’s multiple comparisons test. The data represent the means ± SEM of three independent experiments. ns, no significant difference, * *p* < 0.05, ** *p* < 0.01, *** *p* < 0.001, **** *p* < 0.0001.

## 3. Results and Discussion

### 3.1. Rough Mutant RB14 Infection Reduced Its Intracellular Survival in RAW264.7 Cells and Increased the Death of Macrophages, in Contrast to B. abortus S2308

**Figure 1:**
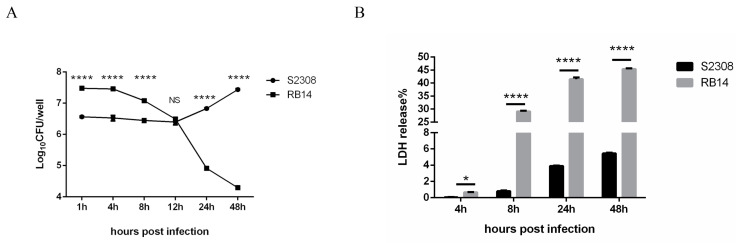
Intracellular growth of *B. abortus* in RAW264.7 macrophages and lactate dehydrogenase (LDH) release into the supernatant of macrophage cultures. (**A**) Intracellular numbers of *B. abortus* RB14 (MOI = 60) and *B. abortus* S2308 (MOI = 1000) in RAW264.7 cells at 1, 4, 8, 12, 24, and 48 h post-infection. (**B**) LDH release measured in supernatants of macrophages after *B. abortus* S2308 (MOI = 1000) and RB14 infection (MOI = 60). Data are means ± SEM of three independent experiments. Data points and error bars represent the means and SEM of triplicate CFU determinations, respectively. NS, no significant difference; * *p* < 0.05, **** *p* < 0.0001.

RAW264.7 cells were infected with *B. abortus* S2308 or RB14, and the intracellular growth of *B. abortus* was assessed at different time points after infection with colony-forming unit (CFU) assays. *B. abortus* S2308 was killed at 0–12 hpi, and the surviving bacteria began to replicate at 12–48 hpi in the cells. However, the intracellular survival of *B. abortus* RB14 decreased after it entered the macrophages (Figure 1A). The supernatants of uninfected, *B. abortus* S2308-infected, and RB14-infected RAW264.7 macrophages were collected at different time points, and the amounts of LDH released from the macrophages were determined. Macrophages infected with rough mutant RB14 showed a much greater percentage release of LDH than macrophages infected with *B. abortus* S2308 (Figure 1B). These results suggest that rough mutant RB14 infection not only decreased the intracellular survival but also caused higher cell death, compared to the smooth *B. abortus* S2308 infection in RAW264.7 cells.

### 3.2. Identification of DEGs in Macrophages Infected with B. abortus S2308 and Its Rough Mutant RB14

**Figure 2:**
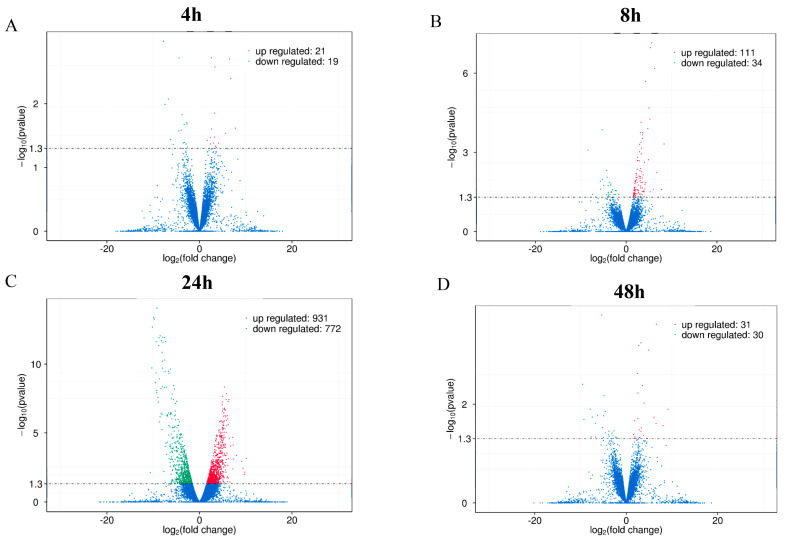
Volcano plots of differentially expressed genes in RAW264.7 cells infected with *B. abortus* RB14 or *B. abortus* S2308. Differentially expressed genes in RAW264.7 cells infected with *B. abortus* RB14 or *B. abortus* S2308 at 4 hpi (**A**), 8 hpi (**B**), 24 hpi (**C**), and 48 hpi (**D**). Red dots indicate upregulated mRNAs, green dots indicate downregulated mRNAs, and blue dots indicate mRNAs with no significant changes.

Based on the intracellular survival results, we designed a comparative transcriptomic analysis of macrophages infected with *B. abortus* S2308 and its rough mutant RB14 to illustrate the underlying pathogenic genes of *B. abortus*. Deep sequencing analyses were performed at 4, 8, 24, and 48 hpi. Each library contained approximately 12–15 G raw reads. After the low-quality reads were removed, approximately 80–96 million clean reads from each sample met the criteria for high-quality eukaryotic transcriptome reconstruction, which requires more than 10 million reads to identify new genes. The Q30 score indicated that more than 90% of the reads from all the samples possessed a 99.8% accuracy rate for the sequenced bases (Table 1). Under these criteria, 40, 145, 1703, and 61 DEGs were identified in RAW264.7 cells after infection with rough mutant RB14 or *B. abortus* S2308 at 4, 8, 24, and 48 hpi, respectively. Compared with their expression in RAW264.7 cells after *B. abortus* S2308 infection, 21, 111, 931, and 31 genes were upregulated in RAW264.7 cells at 4, 8, 24, and 48 h after rough mutant RB14 infection, respectively; and 19, 34, 772, and 30 genes were downregulated after *B. abortus* S2308 infection, respectively (Figure 2); the data were normalized to untreated mock controls. To confirm these transcriptome data, we randomly selected 10 genes for real-time PCR analysis (upregulated genes: *Edn1*, *Plk2*, *Psgt2*, *Phlda1*, *Ccl3*, and *Tnf*; downregulated genes: *Prdx5*, *Hgf*, *Plaur*, and *Pdp1*). The results showed that six of the genes were upregulated and four were downregulated, which is consistent with the transcriptome data (Table 2).

### 3.3. GO Analysis of Differential Host Gene Expression after Infection with Rough Mutant RB14 and B. abortus S2308

**Figure 3:**
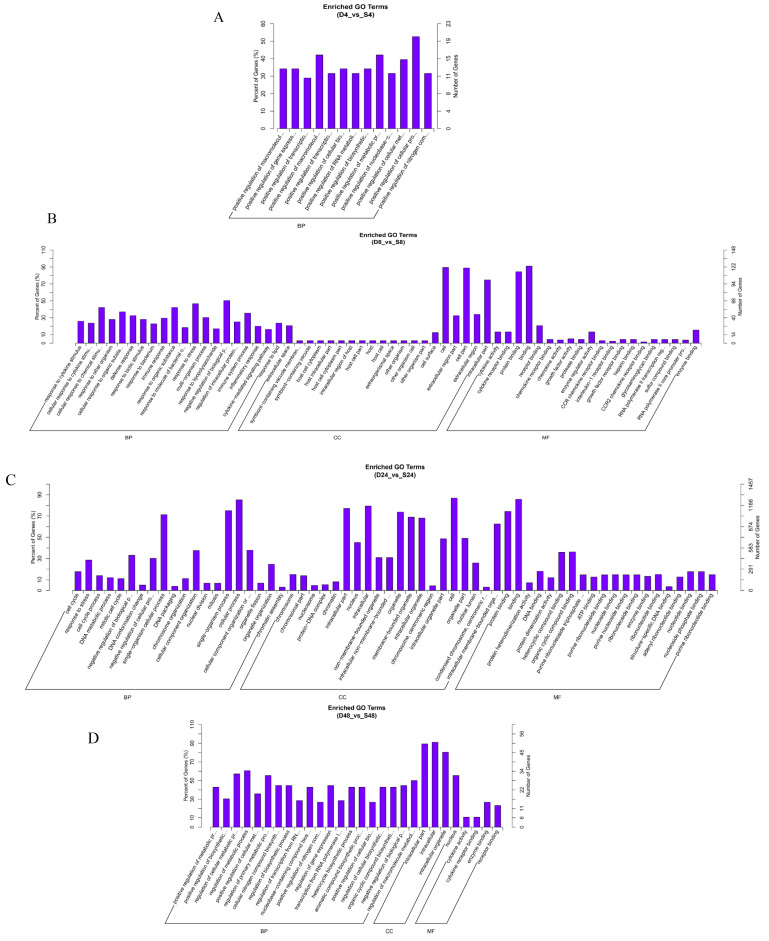
Comparison of biological gene ontology (GO) enrichment of the differentially expressed genes in RAW264.7 cells infected with *B. abortus* RB14 or *B. abortus* S2308. GO analysis of genes differentially expressed in RAW264.7 cells infected with *B. abortus* RB14 or *B. abortus* S2308 at 4 hpi (**A**), 8 hpi (**B**), 24 hpi (**C**), and 48 hpi (**D**). The *x*-axis indicates the GO terms in one of three subcategories: BP (biological process), CC (cellular component), and MF (molecular function). The left *y*-axis indicates the percentage of genes (numbers of enriched genes in each term divided by the total number of genes), and the right *y*-axis indicates the number of genes for each term. D: *B. abortus* RB14; S: *B. abortus* S2308.

Many host genes were differentially expressed at 4, 8, 24, and 48 hpi in macrophages infected with rough mutant RB14 and *B. abortus* S2308. The identified genes were related to the immune response, signal transduction, oxidative stress, the inflammatory response, cytokine-mediated signaling pathways, and the regulation of cellular processes (Figure 3). Among the genes differentially expressed post rough mutant RB14 or *B. abortus* S2308 infection, we focused on those functions that are rarely reported or remain poorly understood in infected cells, and investigated the DEGs related to oxidative stress or novel programmed cell death.

### 3.4. Rough Mutant RB14 Caused Stronger Oxidative Stress Than B. abortus S2308

Reactive oxygen species (ROS) are important effector molecules involved in the killing strategies of a number of bacteria [11,12]. However, *Brucella* has evolved multiple strategies to regulate host gene expression in order to resist oxidative stress. Therefore, screening and identifying the host DEGS related to oxidative stress could allow new strategies for intracellular survival during *Brucella* infection to be identified. The expression of numerous genes related to oxidative stress differed in macrophages infected with rough mutant RB14 and those infected with *B. abortus* S2308 (Table 3). The expression of fewer pro-oxidative stress genes and more antioxidative stress genes were induced in macrophages during *B. abortus* S2308 infection than during rough mutant RB14 infection, suggesting that antioxidation plays an important role in the pathogenic process of *Brucella*. Among these pro-oxidative stress genes, *Edn1*, *Plaur*, and *Hnrnpk* were upregulated and *Abcg1* was downregulated in macrophages infected with rough mutant RB14 relative to their expression after *B. abortus* S2308 infection. Several studies have reported that EDN1 stimulates ROS production via the activation of NADPH oxidase (NOX), xanthine oxidase, and mitochondrial cytochrome oxidase [13,14]. ABCG1 upregulation blunts the activity of pro-oxidant NADPH oxidase and the expression of *Nox4*, which encodes one of the NADPH oxidase subunits [15]. Therefore, all these genes are related to NOXs, which produce cellular ROS. Research has shown that NOXs, xanthine oxidase, peroxisomes, cyclooxygenases (COXs), lipoxygenases (LOXs), and cytochrome P450 enzymes are also sources of ROS [16]. Therefore, *B. abortus* S2308 may regulate the expression of those genes related to ROS generation more rigorously than rough mutant RB14, better preventing the production of ROS. The upregulation of several genes related to the mitochondria or the production of mitochondrial ROS, such as *Plk2*, *Plk3*, *Pdp1*, *Pdk2*, *eIF5A*, *Ifit3*, and *Mtfp1*, whose cognate proteins influence the structure or function of the mitochondria, increased the production of ROS in macrophages infected with the rough mutant RB14 relative to that in macrophages infected with *B. abortus* S2308. Other genes involved in the production of ROS may act through different signaling pathways (*Tgm2*, *Ogt*, *Ier3*, *Mmp9*, *Akap12*, *Src3*, etc.). In summary, the genes involved in ROS production were expressed at higher levels in macrophages infected with RB14 compared to S2308. These results shed light on the different roles played by rough mutant RB14 and *B. abortus* S2308 in regulating the expression of oxidative-stress-related genes during infection of their hosts.

Genes encoding several macrophage antioxidant enzymes (*Prdx5*, *Hmox1*, *Srxn1*, and *Grx1*) showed higher expression during *B. abortus* S2308 infection than during infection with rough mutant RB14. PRDX5, HO-1, SRXN1, and GRX1 are antioxidant enzymes involved in reducing oxidative stress, and can eliminate the ROS produced in host cells. Several genes whose cognate proteins regulate the expression of antioxidant enzymes were also detected, including *Hgf*, *Met*, *Csde1*, *Ccl5*, and *Hao1*. In contrast to infections of rough mutant RB14, the expression of these genes was also upregulated during *B. abortus* S2308 infection, reducing the production of ROS. For example, CCl5 increased GPX1 expression and reduced intracellular ROS levels, which subsequently increased cell survival in both primary neuron cultures and an overexpression model in SHSY5Y cells [17]. HGF/MET signaling upregulates the expression of antioxidant enzymes in cardiomyocytes through the p38α pathway [18]. Therefore, in contrast to rough mutant RB14, *B. abortus* S2308 may upregulate the expression of host antioxidant enzymes and related genes to abolish the production of ROS, which would allow smooth *Brucella* to evade the killing effects of ROS. Compared with rough mutant RB14, *B. abortus* S2308, on the one hand, reduces the expression of the ROS source genes to inhibit the production of ROS, and on the other hand, upregulates antioxidant genes and related genes to eliminate any ROS produced. In summary, the levels of ROS induced in macrophages infected with *B. abortus* S2308 were much lower than those induced in macrophages infected with rough mutant RB14, and oxidative-stress-related genes were also differentially expressed in cells infected with these two strains. These results shed light on the different roles played by rough mutant RB14 and *B. abortus* S2308 in regulating the expression of oxidative-stress-related genes during infection of their hosts.

### 3.5. Rough Mutant RB14 Induced More Expression of Ferroptosis-Associated Genes Than B. abortus S2308

A previous study showed that rough *Brucella* infections induce macrophage apoptosis and pyroptosis, mediated by caspase 2 [19,20], whereas smooth *Brucella* inhibits host cell apoptosis and promotes its own survival and replication in host cells [21,22]. Our transcriptomic analysis demonstrated that several genes related to apoptosis showed much higher expression during infection with rough mutant RB14 than during *B. abortus* S2308 infection (Table S1). More importantly, apart from DEGs related to oxidative stress, we also detected several DEGs involved in the novel programmed cell death, ferroptosis (Table 4). Ferroptosis is an iron-dependent form of necrotic cell death, characterized by oxidative damage to phospholipids [23]. Iron toxicity, lipid peroxidation, and reduced glutathione (GSH) are the pivotal biological features of ferroptosis [24]. Interestingly, our transcriptomic analysis detected several DEGs involved in iron overload, lipid peroxidation, and reduced GSH. Our research data showed that during rough mutant RB14 infection, the expression of the iron-related genes *Fth1* and *Lcn2* was downregulated relative to their expression during *B. abortus* S2308 infection. *Fth1* encodes ferritin heavy chain 1, which plays an important role in the maintenance of the cellular iron balance during ferroptosis [25]. Lipocalin 2 (LCN2) is a critical iron regulatory protein that regulates iron homeostasis under physiological and inflammatory conditions [26,27]. Therefore, macrophages infected with rough mutant RB14 may induce a greater concentration of free intracellular iron than *B. abortus* S2308-infected macrophages due to the downregulation of pivotal iron-associated genes. Our results also showed that during rough mutant RB14 infection, the expression of the lipid-peroxidation-related genes *Cox4i2* and *Alox5* was upregulated relative to their expression in *B. abortus* S2308-infected macrophages. COX4I2 is considered to increase COX activity, thereby promoting ROS production and levels of MDA, a product of lipid peroxidation [28]. ALOX5 is a crucial enzyme that mediates lipid peroxidation by producing lipid peroxides [29]. During rough mutant RB14 infection, the expression of the lipid-peroxidation-related genes *Prdx5* and *Hmox1* in macrophages was downregulated relative to their expression in macrophages during *B. abortus* S2308 infection. Research has shown that cytosolic human peroxiredoxin 5 protects yeast cells from the cytotoxicity and lipid peroxidation caused by paraquat [30]. HMOX1 is an antioxidative gene that also reduces the levels of lipid peroxides and MDA [31]. The expression of the GSH-related genes *Grx1* and *Slc7a11* in macrophages was downregulated during infection with rough mutant RB14 relative to their expression during *B. abortus* S2308 infection. GSH is an important antioxidant with an essential role in maintaining redox homeostasis [32]. Glutaredoxin 1 (GRX1) acts as the main deglutathionylation enzyme and plays a key role in redox signaling and redox homeostasis [33]. SLC7A11 is the light chain subunit of the system x_c_^−^ cystine/glutamate antiporter and plays critical roles in cystine uptake, glutathione synthesis, and ferroptosis resistance [34,35]. In the present study, the expression of transformation-related protein 53 (TRP53), which acts as an upstream negative regulator of SLC7A11, was also increased during rough mutant RB14 infection relative to its expression in *B. abortus* S2308-infected macrophages. Therefore, these up- and downregulated DEGs involved in lipid peroxidation may help rough *Brucella* induce macrophage ferroptosis after infection. In summary, the expression of ferroptosis-associated genes increased in macrophages infected with rough mutant RB14 relative to that in *B. abortus* S2308-infected macrophages.

### 3.6. Rough Mutant RB14 Induced Macrophage Ferroptosis Soon after Infection

**Figure 4:**
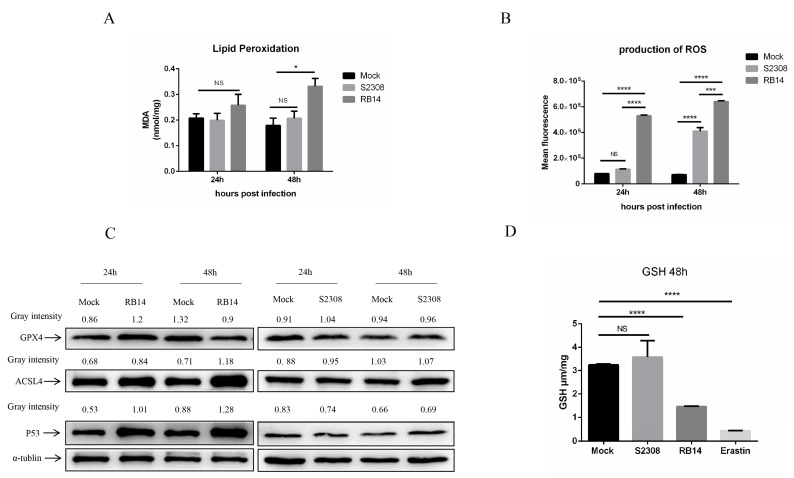
Rough mutant RB14-induced cell death through ferroptosis. (**A**) Lipid peroxidation levels in RAW264.7 cells infected with *B. abortus* RB14 (MOI = 5) and *B. abortus* S2308 (MOI = 100). (**B**) Flow-cytometric analysis of ROS production in RAW264.7 cells infected with *B. abortus* RB14 and *B. abortus* S2308. (**C**) Western blotting analysis of the levels of ferroptosis-related proteins in RAW264.7 cells infected with *B. abortus* RB14 and *B. abortus* S2308. α-tublin was used as the loading control. (**D**) GSH levels in RAW264.7 cells infected with *B. abortus* RB14 and *B. abortus* S2308 (48 hpi). Erastin (10 μM) is a ferroptosis inducer. Data are means ± SEM of three independent experiments. Data were analyzed with two-way ANOVA. NS, no significant difference; * *p* < 0.05, *** *p* < 0.001, **** *p* < 0.0001.

**Figure 5:**
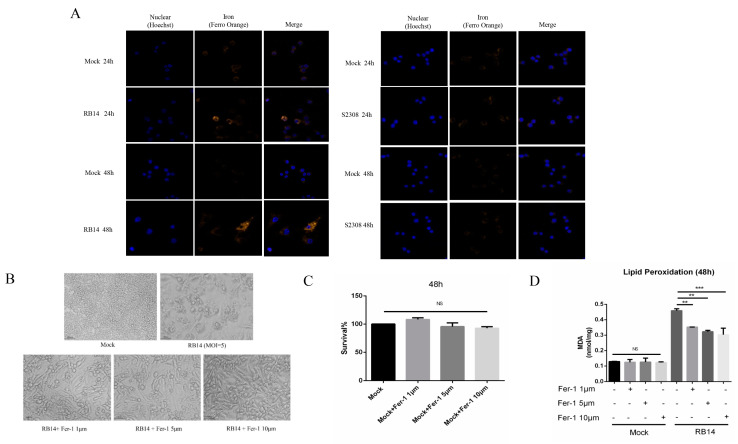
Inhibiting ferroptosis with a ferroptosis inhibitor reduced the death of macrophages. (**A**) Analysis of Fe^2+^ levels in *B. abortus* RB14- and *B. abortus* S2308- infected RAW264.7 cells with the fluorescent probe FerroOrange (orange). (**B**) Cell death induced by RB14 at 48 hpi in macrophages untreated or treated with Fer-1 (bars, 100 µm). (**C**) Toxicity of ferrostatin-1 in cells after 48 h was observed with a cell viability assay. (**D**) Lipid peroxidation levels in *B. abortus* RB14- infected RAW264.7 cells at 48 hpi after treatment with or without ferrostatin-1 (Fer-1); MOI = 5. Ferrostatin-1, an inhibitor of ferroptosis, was added to the macrophage cultures after infection until the indicated times. Data are means ± SEM of three independent experiments. NS, no significant difference; ** *p* < 0.01, *** *p* < 0.001.

Based on our transcriptome data, we speculate that *Brucella* rough mutant RB14 infection may induce macrophage ferroptosis soon after infection. Dixon et al., showed that ferroptotic cell death depends on intracellular iron overload, elevated ROS, and lipid peroxidation [36]. Therefore, we investigated several key hallmarks of ferroptosis during *Brucella* infection. Interestingly, higher levels of lipid peroxidation and ROS were observed in macrophages after infection with rough mutant RB14 than after infection with *B. abortus* S2308 (Figure 4A,B). The level of intracellular labile iron also increased after rough mutant RB14 infection (Figure 5A). Research has shown that GPX4 is a central and essential regulator of ferroptosis. It utilizes its GSH substrate to detoxify lipid peroxidation and plays an essential role in inhibiting ferroptosis [32,37]. The inactivation or depletion of GPX4 induces ferroptosis in a variety of cell types [32,38]. Therefore, we examined the expression of GPX4 in macrophages after rough mutant RB14 infection. Consistent with this concept, we observed that GPX4 expression was reduced in macrophages infected with rough mutant RB14, as measured with western blotting (Figure 4C). We also examined the levels of GSH, the substrate of GPX4. Consistent with the expression of GPX4, the level of GSH decreased after infection with the rough mutant RB14 (Figure 4D). The expression of several other pivotal ferroptosis-related genes and ferroptosis-regulated genes also showed significant changes after rough mutant RB14 infection, including *Acsl4* and *Trp53*, indicating that macrophages infected with rough mutant RB14 underwent ferroptosis (Figure 4C). To more directly examine the involvement of ferroptosis during *Brucella* infection, we tested the effect of the ferroptosis inhibitor ferrostatin-1 (Fer-1) on the macrophage death caused by rough mutant RB14 infection. The addition of Fer-1 to RB14-infected macrophage cultures dose-dependently inhibited cell death (Figure 5B). We then confirmed that the addition of Fer-1 at 1–10 µM had no significant toxic effect on the macrophages (Figure 5C). Furthermore, the lipid peroxidation was also reduced following the addition of Fer-1 at 1–10 µM (Figure 5D). These observations indicate that macrophages infected with rough mutant RB14 undergo ferroptosis. During *B. abortus* S2308 infection, although the production of ROS also increased, lipid peroxidation, GSH, and the expression of the pivotal protein GPX4 showed no significant changes (Figure 4A–D). Further, the level of intracellular labile iron also showed no change after *B. abortus* S2308 infection (Figure 5A), so *B. abortus* S2308 did not induce ferroptosis at 24 or 48 hpi. In summary, rough mutant RB14 induced macrophage ferroptosis early after infection, whereas *B. abortus* S2308 showed no significant increase in the major characteristics associated with ferroptosis in the early period after infection.

Brucellosis is a serious zoonotic infectious disease caused by *Brucella*. It is epidemic throughout the world, and poses a serious threat to public health and livestock development in many countries [39]. Most of the pathogenic mechanisms of *Brucella* involve blocking immune receptors, inhibiting phagolysosome fusion and apoptosis, and reducing antigen presentation [40,41]. However, how *Brucella* manipulates the oxidative stress and related biological processes of its host remains poorly understood. In this study, we undertook a comparative transcriptomic analysis of the RAW 264.7 cell line infected with *B. abortus* S2308 or rough mutant RB14 at different time points after infection to investigate the underlying pathogenic mechanisms of *B. abortus*. We focused on those genes related to oxidative stress and ferroptosis in macrophages.

Endogenous oxidative stress is a consequence of life in an aerobic environment, and bacteria’s interactions with their hosts’ immune systems induce exogenous oxidative stress [42]. ROS are thought to act via two distinct pathways: causing oxidative damage to bacterial biocompounds and affecting the signaling control of cytokines, autophagy, and apoptosis [43]. To overcome the deleterious effects of oxidative damage, *Brucella* has evolved protective, detoxification, and repair mechanisms, including the production of catalase and superoxide dismutase, which directly detoxify ROS, or the production of enzymes that repair oxidative damage to bacterial cellular components [44]. *Brucella* has also evolved several mechanisms to influence the expression of host genes, which reduce the production of ROS and oxidative damage. In a previous study, we demonstrated that several host genes are regulated by *B. abortus* to reduce the production of ROS and facilitate *Brucella* survival [45,46,47]. However, the host oxidative-stress-related genes regulated by *Brucella* are not well understood, and further genes and regulatory mechanisms must be identified. The main cellular source of ROS is the mitochondrial respiratory chain and the family of NOXs [48,49]. Other sources of ROS in macrophages include xanthine oxidase, peroxisomes, COXs, LOXs, and cytochrome P450 enzymes [50]. The cellular antioxidant defense system balances the production of ROS against the production of antioxidant enzymes, such as the superoxide dismutases (SODs), GSH, and coenzyme Q10 [16]. The results of the present study show that both antioxidative stress and pro-oxidative stress effects correlated with these gene expression profiles. Several pro-oxidative stress genes (*Edn1*, *Plaur*, *P2X7*, and *Hnrnpk*) related to NOXs were upregulated in macrophages infected with rough mutant RB14 relative to their expression in *B. abortus-*S2308-infected macrophages. Moreover, *Alox5*, *Cox4i2*, and *Ptgs2*, which encode proteins that facilitate the production of more ROS, were also upregulated in macrophages infected with rough mutant RB14 relative to their expression in *B. abortus*-S2308-infected macrophages. These genes are related to other pro-oxidative enzymes, such as COXs, LOXs, and cytochrome P450 enzymes. It is therefore possible that in contrast to rough mutant RB14, smooth *B. abortus* inhibits the expression of these pro-oxidative genes to reduce the production of ROS. Genes encoding several host antioxidant enzymes or related proteins (*Prdx5*, *Hmox1*, *Srxn1*, *Grx1*, *Hgf*, *Met*, *Csde1*, *Ccl5*, and *Hao1*) showed higher expression during *B. abortus* S2308 infection than during rough mutant RB14 infection. These antioxidant enzymes reduce the production of ROS, indicating that *B. abortus* may reduce ROS production by upregulating the expression of antioxidant enzymes or related genes. This hypothesis is consistent with a report that *Brucella* does not induce an oxidative burst when it invades its host cells [51]. Therefore, our comparative transcriptomic analysis of the differential expression of oxidative stress genes between *B. abortus* S2308 and rough mutant RB14 should help us to characterize the underlying pathogenic mechanism during *B. abortus* infection.

Previous studies have shown that smooth *Brucella* inhibits macrophage apoptosis, whereas rough *Brucella* induces macrophage apoptosis, pyroptosis, and oncosis [19,22,52,53]. Consistent with those reports, we have demonstrated that rough mutant RB14 caused much greater LDH release from macrophages than *B. abortus* S2308. We also showed that several DEGs related to apoptosis were more expressed during rough mutant RB14 infection than during *B. abortus* S2308 infection (Table S2). More importantly, our results indicate for the first time that ferroptosis is also involved in rough mutant RB14 infection. Ferroptosis is an iron-dependent form of regulated cell death caused by unrestricted lipid peroxidation and subsequent membrane damage [36]. Research has shown that two key initial signals trigger ferroptosis, excessive iron accumulation and the inhibition of GPX4, a protein that prevents lipid peroxidation and detoxifies lipid hydroperoxides [54].

The expression of iron-related genes *Fth1* and *Lcn2* was downregulated in macrophages after rough mutant RB14 infection relative to their expression post *B. abortus* S2308 infection. FTH1 is the heavy chain of ferritin, a widely expressed and highly conserved protein, consisting of two types of polypeptide chain: the ferritin heavy chain and ferritin light chain. The ferritin heavy chain catalyzes the Fe^2+^ oxidation reaction, whereas the ferritin light chain plays an important role in the storage of Fe^3+^. Both chains are essential for the maintenance of iron homeostasis and the prevention of iron overload. FTH1 has also been shown to inhibit ferroptosis through ferritinophagy in Parkinson’s disease [55]. Therefore, lower expression of *Fth1* may accelerate iron accumulation. *Lcn2* encodes lipocalin 2, which regulates iron homeostasis and prevents the bacterial reuptake of iron-loaded siderophores [56]. LCN2 also plays a pivotal role in the inhibition of ferroptosis. LCN2 expression blocks ferroptotic cell death by reducing iron accumulation and subsequent oxidative damage. Therefore, the downregulation of *Lcn2* may facilitate ferroptosis by increasing iron accumulation.

**Figure 6:**
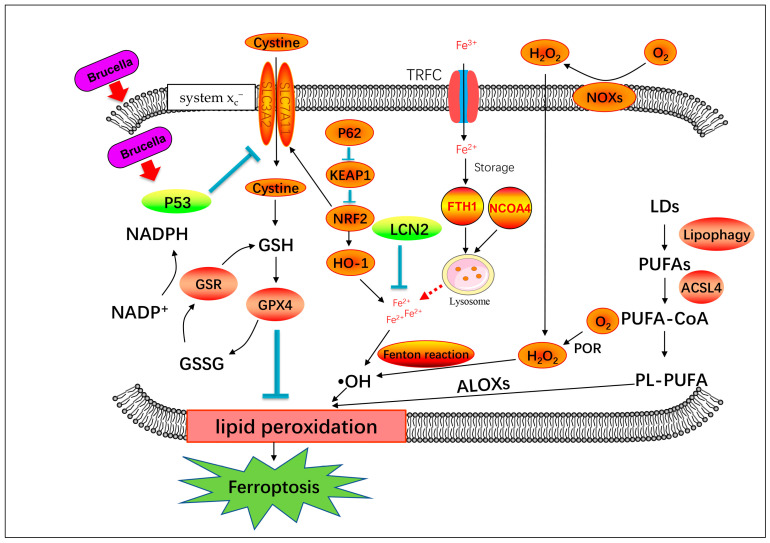
Schematic diagram of genes involved in ferroptosis process.

During lipid peroxidation, the high levels of polyunsaturated fatty acids (PUFAs) in cellular and organellar membranes are damaged by ROS, and this is an important index of ferroptosis. We identified several DEGs involved in ferroptosis and lipid peroxidation, including *Cox4i2*, *Alox5*, *Prdx5*, *Slc7a11*, and *Grx1*. Infection with rough mutant RB14 upregulated lipid-peroxidation-related genes *Alox5* and *Cox4i2* in macrophages relative to their expression after infection with *B. abortus* S2308. Research has shown that ROS-mediated lipid peroxidation is mainly attributable to ALOX [57]. Regulating the activity of ALOX5 has been shown to be a useful way to control ROS- and ferroptosis-induced damage, which promotes degeneration in retinal diseases [58]. COX4I2 is also reported to increase ROS production and lipid peroxidation, leading to ferroptosis [28]. Infection with rough mutant RB14 also suppressed the expression of several antioxidant genes (*Prdx5*, *Slc7a11*, and *Grx1*) in macrophages, in contrast to infection with *B. abortus* S2308. Our data indicate that macrophages infected with rough mutant RB14 showed greater lipid peroxidation at 24 hpi than those infected with *B. abortus* S2308. Therefore, it is possible that rough mutant RB14 infection induces ferroptosis at 24 hpi, whereas *B. abortus* S2308 does not. To confirm whether rough mutant RB14 will induce ferroptosis, we investigated the pivotal biological characteristics of ferroptosis in macrophages with infection of the rough RB14 mutant. The production of ROS and lipid peroxidation increased at 24 and 48 h after infection with rough mutant RB14. Iron accumulation in macrophages was also observed with immunofluorescence after rough mutant RB14 infection. The expression of several pivotal genes associated with ferroptosis (*Gpx4*, *Acsl4*, *Trp53*, *Slc7a11*, and *Nox2*) also changed after rough mutant RB14 infection. GPX4 is a glutathione- and selenium-dependent glutathione peroxidase that can detoxify lipid hydroperoxides. Research has shown that GPX4 inhibitors (e.g., RSL3, ML162, ML210, FIN56, and FINO2) are also classic ferroptosis activators [59]. Acyl-CoA synthetase long-chain family member 4 (ACSL4) plays a key role in promoting ferroptosis by incorporating PUFAs into cellular phospholipids [60], because PUFAs are the main substrates of lipid peroxidation in the process of ferroptosis, which damages membrane structure and function [54]. Therefore, GPX4 and ACSL4 regulating lipid peroxidation might influence the ferroptosis in macrophages with rough mutant RB14 infection. A previous study showed that the P53–system x_c_^−^–GSH–GPX4 signaling pathway is a main route through which ferroptosis is induced [10]. System x_c_^−^ is composed of two protein subunits, SLC7A11 and SLC3A2. SLC7A11 is an amino acid transporter that imports cystine and exports glutamate [35]. The expression of SLC7A11 and the importation of cystine promote GSH biosynthesis and ferroptosis resistance [36]. GSH is commonly referred to as the body’s main antioxidant. It is necessary for the activity of GPX4, a selenoenzyme that functions as a central ferroptosis repressor by reducing toxic phospholipid hydroperoxides to nontoxic phospholipid alcohols [61]. Interestingly, our results not only show that the expression of GPX4 and SLC7A11 decreased, but also that the level of GSH was downregulated in macrophages after infection with rough mutant RB14. The expression of the upstream transcription factor P53 also increased after infection with rough mutant RB14. Research has shown that the transcription of P53 may affect the expression of the SLC7A11 protein and thus influence the production of reduced GSH [62]. Therefore, it is possible that the P53–system x_c_^−^–GSH–GPX4 signaling pathway is involved in the macrophage ferroptosis caused by rough mutant RB14. How these ferroptosis related genes interact with each other is presented in Figure 6. According to our data, rough mutant RB14 infection induced ferroptosis, whereas *B. abortus* S2308 induced no obvious ferroptotic characteristics in macrophages. A previous study showed that *Brucella* infection inhibits macrophage apoptosis, promoting the bacterium’s intracellular growth [63]. Does *B. abortus* infection inhibit macrophage ferroptosis to promote its intracellular survival and growth in the early period after infection? This question warrants further investigation, together with studies that clarify the complex functions and regulatory mechanisms involved. Research has shown that ferroptotic stress protects macrophages against intracellular *E. coli*, *Salmonella pullorum*, and *Staphylococcus aureus* [64], whereas other research has shown that *Mycobacterium tuberculosis* infection also induces cell ferroptosis, facilitating mycobacterial spread [24]. Whether ferroptosis in macrophages is an efficient macrophage defense strategy against *Brucella*, or whether ferroptosis in macrophages is a novel strategy that allows *Brucella* to evade the host’s immune response requires clarification. The function of ferroptosis during *Brucella* infection and the underlying regulatory mechanisms also warrant further study.

Overall, our results demonstrate that a large number of novel genes related to oxidative stress and ferroptosis are regulated by *B. abortus*, which should allow us to identify further immunity-evading strategies used by *Brucella*. More importantly, this study showed that ferroptosis, a novel type of programmed cell death, is involved in infection by rough *Brucella*. This knowledge should extend our understanding of the pathogenic mechanisms of *Brucella*.

System Xc^−^mediated cystine uptake and subsequent GSH production and GPX4 activation may be involved in *Brucella*-induced ferroptosis. The generation of polyunsaturated phospholipids (by ACSL4 and LPCAT3) and subsequent activation of ALOX may have a main role in promoting lipid peroxidation. This process requires hydrogen peroxide (H_2_O_2_) production from an iron-mediated Fenton reaction or the activation of POR, NOX, P62/KEAP1/NRF2/HO-1, or other pathways.

## 4. Conclusions

Our study revealed that the expression of numerous host pro-oxidative stress genes was downregulated in *B. abortus* S2308-infected macrophages at 4 and 8 hpi relative to their expression in rough mutant RB14, most of which encode oxidases or oxygenases. At 24 and 48 hpi, *B. abortus* S2308 upregulated the expression of the host’s antioxidative stress genes, especially several genes encoding antioxidant enzymes. Our results also demonstrate for the first time that ferroptosis is involved in rough *B. abortus* infection. While *B. abortus* S2308 infection did not induce obvious ferroptosis at an early time post-infection. The key findings, that *Brucella* manipulates oxidative stress and ferroptosis during infection for its intracellular survival, will help us to elucidate the pathogenicity of *B. abortus*. However, the mechanism by which different phenotype strains of *Brucella* regulate ferroptosis and the function of ferroptosis on infection need to be further clarified, which will help us to illustrate the pathogenicity of *B. abortus*.

## Figures and Tables

**Table 1:** Statistics for RNA-seq datasets.

Sample	Raw Reads	Clean Reads	Clean Bases	Q20	GC Content	Error Rate (%)
S4	91,790,708	84,130,842	12.62 G	96.39	47.31	0.02
S8	92,109,518	88,440,564	13.27 G	96.94	47.23	0.02
S24	89,970,192	86,345,302	12.95 G	96.79	47.98	0.02
S48	88,759,706	84,500,112	12.68 G	96.45	47.19	0.02
D4	93,288,264	84,880,934	12.73 G	96.54	47.25	0.02
D8	99,077,544	95,205,488	14.28 G	96.83	47.4	0.02
D24	90,294,254	86,709,478	13.01 G	96.9	47.33	0.02
D48	87,797,844	83,468,526	12.52 G	96.67	47.59	0.02

D: *B. abortus* RB14, S: *B. abortus* S2308.

**Table 2:** Verification of transcriptome data with real-time RT-PCR (RB14 versus S2308).

Gene	Transcriptome Data		Real-Time RT-PCR
	log_2_ (Fold Change)	*p*-Value	Fold Change
*EDN1* (4 H)	3.29317	0.0140227	1.94
*PLK2*	1.67528	0.037466	3.43
*TNF* (8 H)	2.4	0.00925408	14.62
*PLK3*	2.42564	0.013572	5.69
*PHLDA1*	2.96505	0.000950341	14.82
*CCL3*	2.1174	0.0447028	10.41
*PDP1*	−5.66545	0.0125231	0.56
*PRDX5* (24 H)	−2.76565	0.00357265	0.49
*PLAUR*	−2.50673	0.002	0.61
*HGF*	−4.73832	0.010	0.46

**Table 3:** Oxidative-stress-related genes significantly differentially expressed post-infection (RB14 versus S2308).

Genes	log_2_ (Fold Change)	*p*-Value	Description
**Response to oxidative stress (4 h)**			
*HNRNPK*	Inf	0.0322088	Heterogeneous nuclear ribonucleoprotein-K
*EIF5A*	3.32074	0.0026443	Translation elongation factor, IF5A
*EDN1*	3.29317	0.0140227	Endothelin-1
*PLK2*	1.67528	0.037466	Polo-like kinase 2
*MADD*	−7.44332	0.0102399	dDENN domain
*HAX1*	−3.75837	0.0148648	HS1-associating protein X-1
**Response to oxidative stress (8 h)**			
*EDN1*	5.24481	5.52 × 10^−5^	Endothelin-1
*PTGS2*	3.60541	0.000294431	Haem peroxidase
*PLK2*	2.97997	0.000497573	Polo-like kinase 2
*PLK3*	2.42564	0.013572	Polo-like kinase 3
*PLAUR*	2.04848	0.0122223	Urokinase plasminogen activator surface receptor
*PDP1*	−5.66545	0.0125231	Pyruvate dehydrogenase phosphatase 1
**Response to oxidative stress (24 h)**			
*COX4I2*	4.67318	0.0440457	Complex IV subunit 4 isoform 2
*MTFP1*	3.64855	0.0250454	Mitochondrial fission process 1
*SESN1*	3.06352	0.00987441	AhpD-like II Sestrin
*SLC8A1*	2.98886	0.0471702	Sodium/calcium exchanger 1
*PARP1*	2.37893	0.00630785	Poly [ADP-ribose] polymerase 1
*ALOX5*	2.04461	0.0462303	Lipoxygenase
*AOX2*	−2.89137	0.0266167	Aldehyde oxidase
*SLC7A11*	−8.50415	6.94 × 10^−7^	L-type amino acid transporter
*MET*	−6.95446	4.77 × 10^−5^	Hepatocyte growth factor receptor
*TGM2*	−6.30984	7.52 × 10^−5^	Transglutaminase-2
*AKAP12*	−4.89105	0.000236997	A-kinase anchor protein 12
*HGF*	−4.73832	0.0100114	Hepatocyte growth factor
*IFIT3*	−4.22289	2.16 × 10^−5^	Interferon-induced protein with tetratricopeptide repeats 3
*ERCC1*	−3.92249	0.00065049	DNA excision repair protein ERCC-1
*SRXN1*	−3.6182	0.0001	Sulfiredoxin
*SRC*	−3.43942	0.0263683	Protein kinase domain
*HAO1*	−3.04748	0.0079316	2-Hydroxyacid oxidase 1
*IER3*	−2.92629	0.00043274	Radiation-inducible immediate-early gene IEX-1
*PRDX5*	−2.76565	0.00357265	Peroxiredoxin-5
*GLRX*	−2.69232	0.000932574	Glutaredoxin-1
*OGT*	−2.60033	0.00849352	Tetratricopeptide repeat
*HMOX1*	−2.4394	0.00320755	Heme oxygenase 1
*MMP9*	−2.35686	0.0320668	Matrix Metalloproteinase 9
*NFKB1*	−1.73443	0.0462194	NF-kappa-B/Dorsal
**Response to oxidative stress (48 h)**			
*DNM2*	4.92666	0.000780525	Dynamin, GTPase domain
*NR4A1*	2.5619	0.0196435	Nuclear hormone receptor
*HERC2*	2.83	0.032	HECT domain and RCC1-like domain 2
*CSDE1*	-inf	0.00981001	Cold shock protein
*ABCG1*	−2.0581	0.0466713	ATP-binding cassette transporter G1

**Table 4:** Ferroptosis-related genes significantly differentially expressed at 24 h post-infection (RB14 versus S2308).

Genes	log_2_ (Fold Change)	*p*-Value	Description
Ferroptosis			
*MET*	−6.95446	4.77 × 10^−5^	Hepatocyte growth factor receptor
*COX4I2*	4.67318	0.0440457	Complex IV subunit 4 isoform 2
*ALOX5*	2.04461	0.0462303	Lipoxygenase 5
*PRDX5*	−2.76565	0.00357265	Peroxiredoxin-5
*HMOX1*	−2.4394	0.00320755	Heme oxygenase 1
*SLC7A11*	−2.50673	0.002	L-type amino acid transporter
*TRP53*	5.08989	0.0124723	p53 tumor suppressor
*FTH1*	−2.91328	0.0137136	Ferritin heavy chain
*LCN2*	−6.2905	0.00278457	Lipocalin 2

## Data Availability

The raw data that support the findings of this study are available from National Center for Biotechnology Information at https://www.ncbi.nlm.nih.gov/sra/PRJNA905888 (accessed on 17 January 2023), reference number: PRJNA905888.

## Supplementary Materials

The following supporting information can be downloaded at: https://www.mdpi.com/article/10.3390/pathogens12101189/s1, Table S1: Significantly regulated apoptosis-related genes postinfection (RB14 versus S2308); Table S2: Specific primers of candidate genes for RT-qPCR used in this study.
