# Supplementary material for: Brucella abortus Rough-Type Mutant Induces Ferroptosis and More Oxidative Stress in Infected Macrophages
Source: Pathogens. 2023 Sep 23;12(10):1189. doi: 10.3390/pathogens12101189 (PMC10609801; doi:10.3390/pathogens12101189)
Supplement: Supplementary file 1 [file pathogens-12-01189-s001.zip › pathogens-2537991-supplementary.pdf]

Table S1. Significantly regulated apoptosis-related genes postinfection (RB14 versus S2308)

Gene	log2(foldchange)	P value	description
**Apoptosis regulators (4h)**			
Madd	-7.44332	0.0102399	MAP kinase-activating death domain protein
Hax1	-3.75837	0.0148648	HS1-associating protein X-1
Hnrnpk	inf	0.0322088	heterogeneous nuclear ribonucleoprotein-K
Zmynd11	-3.89904	0.0259966	Zinc finger MYND domain-containing protein 11
Eif5a	3.32074	0.0026443	Translation elongation factor, IF5A
Edn1	3.29317	0.0140227	Endothelin-1
Plk2	1.67528	0.037466	Polo-like kinase 2
**Apoptosis regulators (8h)**			
Tnf	2.4	0.00925408	Tumour necrosis factor
Phlda1	2.96505	0.000950341	Pleckstrin homology-like domain family A member 1
Ifit2	1.84511	0.0219366	Interferon-induced protein with tetratricopeptide repeats 2
Ptgs2	3.60541	0.000294431	Forkhead box protein L2
Cd40	3.88115	0.0287682	Tumour necrosis factor receptor 5
Ccl3	2.1174	0.0447028	C-C motif chemokine 3
Edn1	3.29317	0.0140227	Endothelin-1
Skil	5.27626	0.0315321	Ski-like protein
Cd24a	-2.36321	0.0279978	Signal transducer CD24
**Apoptosis regulators (24h)**			
Rffl	-2.83177	0.031	Zinc finger, RING-type
Tnfrsf23	-3.32059	0.0003	TNFR/NGFR cysteine-rich region
Bcl2l1	-1.91148	0.031	Apoptosis regulator, Bcl-2 protein
Bcl2a1a	-2.44177	0.047	Bcl-2-related protein A1
Mcl1	-3.94589	0.001	Apoptosis regulator, Mcl-1

Serpine1	-6.58351	4.60E-10	Serpin domain
Serpinb2	-8.05638	1.39E-11	Serpin B2
Melk	3.83023	0.0012424	Maternal embryonic leucine zipper kinase
Serpinb9	-4.48496	5.57E-05	Serpin B9/maspin
Serpinb9b	Inf	0.016	Serpin B9/maspin
Cebpb	-2.93619	0.0004	CCAAT/enhancer-binding protein beta
Brca2	4.2329	0.000949164	Breast cancer type 2 susceptibility protein
E2f2	3.2491	0.00743477	E2F/DP family
E2f1	2.35434	0.0415558	E2F/DP family
Plekhf1	3.45776	0.0246415	Pleckstrin homology domain
Trp53	5.08989	0.0124723	p53 tumour suppressor
Mmp9	-2.35686	0.032	metallopeptidase
Hgf	-4.73832	0.010	Hepatocyte growth
Grina	-2.19038	0.007	Bax inhibitor 1-related
Src	3.43942	0.026	Tyrosine-protein kinase
Tmbim6	-3.76295	0.0006	Bax inhibitor 1, conserved site
Mef2c	3.66429	0.015	MADS-box
Plekhf1	3.45776	0.0246415	Pleckstrin homology domain
Tnfaip3	-2.69206	0.0186418	Tumor necrosis factor alpha-induced protein 3
**Apoptosis regulators (48h)**			
Ambra1	inf	0.0493014	Activating molecule in BECN1-regulated autophagy protein 1
Jade1	8.04701	0.0272346	Protein Jade-1
Inhba	3.90835	0.00946889	Inhibin beta A chain
Herc2	2.83	0.032	HECT domain and RCC1-like domain 2
Nr4a1	2.5619	0.0196435	Nuclear hormone receptor
Tnfrsf1b	1.63388	0.0412559	Tumour necrosis factor receptor 1B
Ncor1	5.005	0.0087	nuclear receptor co-repressor
Arid1a	3.24	0.035	AT-rich interactive domain-containing protein 1A

Table S2. Specific primers of candidate genes for RT-qPCR used in this study

Primers	Sequences
Prdx5-F	GGCTGTTCTAAGACCCACCTG
Prdx5-R	GGAGCCGAACCTTGCCTTC
TNF-α-F	AGACCCTCACACTCAGATCA
TNF-α-R	TTGCTACGACGTGGGCTACA
Plaur-F	CAGAGCTTTCCACCGAATGG
Plaur-R	GTCCCCGGCAGTTGATGAG
Pdp1-F	GGCCCAGACGAATTGGAATC
Pdp1-R	AGTTGAGTTGGTGCTGGCAT
Plk2-F	CCTGCGGACTATCACCTACCA
Plk2-R	CTGCCCATCTTCAGAAGGCT
Plk3-F	CTGTAGCCGAAAGTCCCTGG
Plk3-R	AGGATCTGGCGCAGGTAGTA
Edn1-F	GGCCCAAAGTACCATGCAGA
Edn1-R	GATGGCCTCCAACCTTCGTA
Hgf-F	CCATCCCCTATGCAGAAGGAC
Hgf-R	CTGATACACCTGTTGGCACAC
Phlda1-F	GGGCTACTGCTCATACCGC
Phlda1-R	AAAAGTGCAATTCCTTCAGCTTG
β-actin-F	GGCTGTATTCCCCTCCATCG
β-actin-R	CCAGTTGGTAACAATGCCATGT
